# Supplementary material for: Beneficial Effects of Tacrolimus on Brain-Death-Associated Right Ventricular Dysfunction in Pigs
Source: Int J Mol Sci. 2023 Jun 21;24(13):10439. doi: 10.3390/ijms241310439 (PMC10341891; doi:10.3390/ijms241310439)
Supplement: Supplementary file 1 [file ijms-24-10439-s001.zip › ijms-2409305-supplementary.pdf]

Supplemental Data

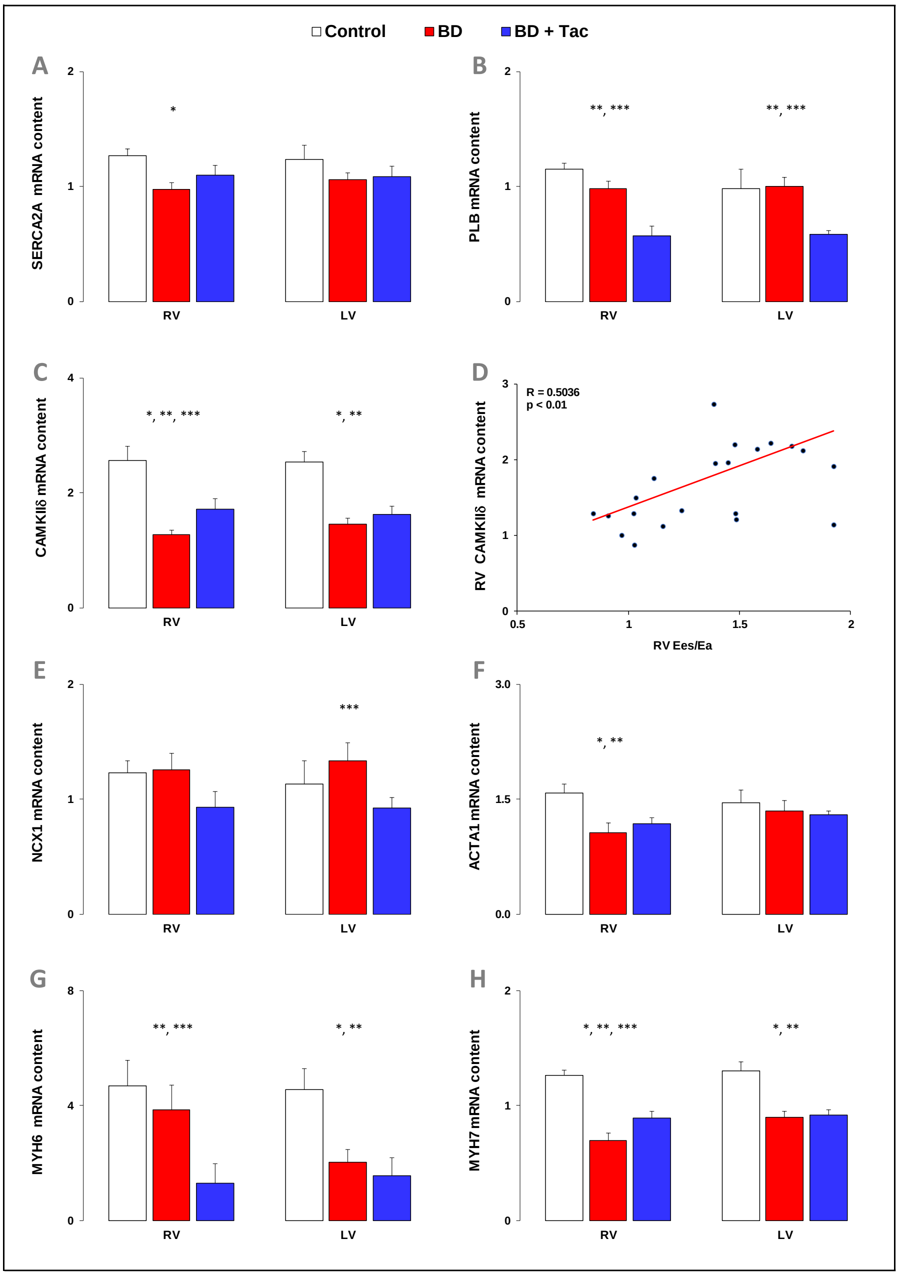

Figure S1. Myocardial expression in brain death–induced right ventricular dysfunction with/without tacrolimus pretreatment. ATPase sarcoplasmic/endoplasmic reticulum Ca2+ transporting 2 (SERCA2A) (A), Phospholamban (PLB) (B), Ca2+/calmodulin-dependent protein kinase II delta (CAMKII) (C), Sodium/calcium exchanger protein (NCX1) (E), Actin alpha 1 (ACTA1) (F), Myosin heavy chain -(MYH) 6 (G) and 7 (H) relative mRNA content in the right (RV) and left ventricles (LV) from the control (n=7; white bars), placebo (brain death, BD; n=9; red bars), and tacrolimus pretreated brain death (BD+Tac; n=8; blue bars) groups 7-hours after the Cushing reflex (CR+7 hours). Values are expressed as mean ± standard error of the mean. * p<0.05 Control vs. BD, ** p<0.05 Control vs. BD+Tac, *** p<0.05 BD vs. BD+Tac.

Correlations between the Ees/Ea ratio and genic expression for (D) in the RV.
